# Implementation and Validation of Constrained Density Functional Theory Forces in the CP2K Package

**DOI:** 10.1021/acs.jctc.2c00284

**Published:** 2022-06-14

**Authors:** Christian S. Ahart, Kevin M. Rosso, Jochen Blumberger

**Affiliations:** † Department of Physics and Astronomy and Thomas Young Centre, University College London, London WC1E 6BT, United Kingdom; ‡ Pacific Northwest National Laboratory, Richland, Washington 99354, United States

## Abstract

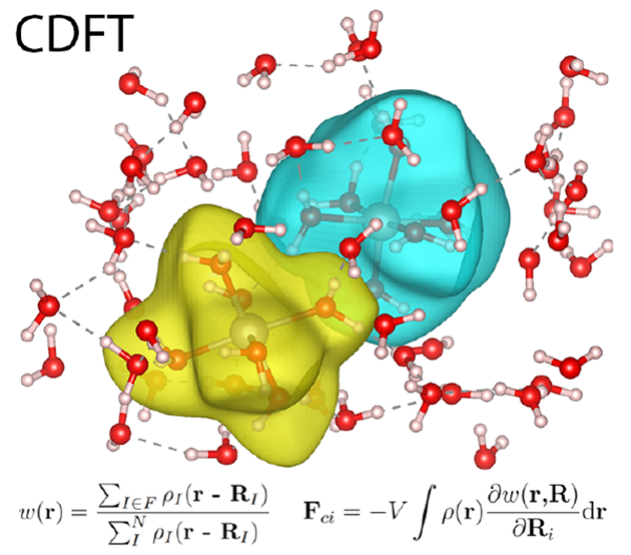

Constrained density functional theory (CDFT) is a powerful tool for the prediction of electron transfer parameters in condensed phase simulations at a reasonable computational cost. In this work we present an extension to CDFT in the popular mixed Gaussian/plane wave electronic structure package CP2K, implementing the additional force terms arising from a constraint based on Hirshfeld charge partitioning. This improves upon the existing Becke partitioning scheme, which is prone to give unphysical atomic charges. We verify this implementation for a variety of systems: electron transfer in (H_2_O)_2_^+^ in a vacuum, electron tunnelling between oxygen vacancy centers in solid MgO, and electron self-exchange in aqueous Ru^2+^–Ru^3+^. We find good agreement with previous plane-wave CDFT results for the same systems, but at a significantly lower computational cost, and we discuss the general reliability of condensed phase CDFT calculations.

## 1 Introduction

The electron self-interaction error is one of the major shortcomings of standard density functionals.^1−3^ While this long-standing problem has been addressed at a fundamental level through development of 1-electron self-interaction error free functionals^4,5^ and, very recently, through neural network machine learning,^6^ several correction schemes for standard functionals have also been developed, including Perdew–Zunger self-interaction correction,^1^ DFT+U,^7^ optimal tuning of range-separated hybrid functionals,^8^ constrained density functional theory (CDFT),^9,10^ its multideterminant extension CDFT configuration interaction (CDFT-CI),^11^ and the localized orbital scaling correction approach (LOSC).^12^

CDFT, on which we focus in the current work, is particularly attractive in the context of electron transfer (ET) calculations. An external potential is added to the Kohn–Sham (KS) Hamiltonian to enforce localization of the excess electron on the electron donor or acceptor, thereby creating a set of charge localized diabatic states that can be used to obtain the basic quantities of ET theories (reorganization energy, driving force, and electronic coupling). The rationale behind CDFT is that the charge localized diabatic states suffer less from the electron delocalization error than the adiabatic electronic states in time-dependent (TD)DFT calculations. This is particularly true at ET transition states where the exact adiabatic ground state is delocalized over donor and acceptor and its energy is strongly underestimated by standard density functionals due to the wrong scaling of these functionals with fractional electron number, resulting in too low ET barriers and strongly overestimated ET rates.^10,11^

In recent years there have been several new implementations of CDFT in popular DFT packages,^13−22^ which generally follow the seminal work by Wu and Van Voorhis.^9^ A Lagrangian multiplier is introduced to search for an external potential applied to the Kohn–Sham Hamiltonian, performed self-consistently with a second iteration loop in addition to that of a standard DFT calculation. The definition of this external potential introduces a weight function, describing the partitioning of electron density (or charge). In their earlier work,^9^ Wu and Van Voorhis utilized the Lowdin atomic population scheme,^23^ later recommending real space partitioning schemes of the electron density, in particular Becke partitioning.^24^ As a purely geometric approach that divides space equally among all atoms, Becke partitioning of the electron density avoids any issues with basis set convergence found for Lowdin or Mulliken atomic charge partitioning.^25^ An alternative real space partitioning scheme is the one according to Hirshfeld^26^ where molecular electron density is assigned to atoms in proportion to their promolecular density, thus accounting for their different sizes.

Table 1 demonstrates the problem of equally dividing space among all atoms as done in Becke partitioning, that for a water molecule the oxygen atom becomes positively charged and the hydrogen atoms become negatively charged. This is in direct contrast to Hirshfeld charge partitioning, which predicts a qualitatively correct charge distribution. The qualitative failure resulting from equal division of space for charge partitioning in heteronuclear systems is well-known,^24^ where it is common to define atomic size adjustments based on either covalent or ionic radii.^18^ Such introduction of empirical parameters is undesirable, with significant ambiguity in their choices.

**Table 1:** Atomic Charges for a Neutral Water Molecule According to Different Partitioning Schemes

atom	Becke	Hirshfeld
O	0.84	–0.30
H	–0.42	0.15

Recently CDFT has been implemented in the CP2K simulation package;^18^ however, the CDFT forces required for CDFT geometry optimization and molecular dynamics simulation are currently only available for Becke partitioning of the electron density. In this work we report the implementation of CDFT forces arising from the more robust Hirshfeld partitioning scheme of the electron density. We benchmark our implementation against previous plane-wave CDFT calculations^13^ also performed using Hirshfeld partitioning, finding good agreement for both geometry optimization and molecular dynamics for electron tunnelling between oxygen defects in MgO^27^ and electron self-exchange in aqueous Ru^2+^–Ru^3+^.^13^

Through considering a wider selection of systems than previous work, we are also able to discuss the general reliability of condensed phase CDFT calculations. With the example of charge transfer in two organic crystals where fully localized polarons do not exist, we demonstrate that a useful diagnostic tool to identify symmetry splitting and the transfer of fractional electrons resulting from unphysical diabatic states is the integrated absolute spin density (IASD)
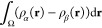
1where ρ_α_(**r**) and ρ_β_(**r**) are the electron densities of the alpha and beta spin channels.

The remainder of the paper is organized as follows. Section 2 summarizes briefly the theory of CDFT and the implementation of the required force terms, followed by results for both CDFT geometry optimization (Section 3.1) and CDFT molecular dynamics (Section 3.2). An example of systems for which CDFT calculations can be problematic is shown in Section 3.1, and a discussion of the general reliability of condensed phase CDFT calculations is presented in Section 3.4. Concluding remarks are made in Section 4.

## 2 Theory and Implementation

CDFT is a well established method, with many recent implementations in popular DFT packages.^15,16,18,19,22^ As such, we choose to only briefly summarize the theory relevant to this work.

Charge or spin localized states are constructed by minimizing the energy functional *E*[ρ] under the condition that the constraint
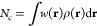
2is satisfied. Here *w*(**r**) is a weight function that defines how electron density is assigned to atoms or molecules in the constraint region, e.g., electron donor and electron acceptor, and *N*_c_ is the constraint value, e.g., the charge or spin of the atoms or molecules, or their charge or spin difference. Both remain fixed during CDFT minimization.

The constrained minimization is performed by introducing a Lagrangian multiplier *V* and a new energy functional
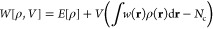
3

*W*[ρ, *V*] is minimized with respect to ρ for a given *V*, and *V* is iteratively adjusted so that the minimized electron density obeys the constraint eq 2.

In CDFT the total force on an atom *i* is given by
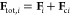
4where **F**_*i*_ is the usual force arising from the unmodified DFT functional *E*[ρ] and **F**_c*i*_ is the additional force arising from the constraint. The latter is given by
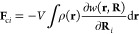
5

The Hirshfeld weight function is constructed from the promolecular atomic densities ρ_*i*_(**r – R**_*i*_) = ρ_*i*_(*r*) where *r* = |**r – R**_*i*_|. For a system with *N* total atoms and a charge difference constraint defined between donor atoms *D* and acceptor atoms *A*, the weight function has the form
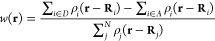
6

The derivative of the weight function can be shown to be^13^
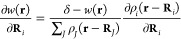
7where
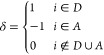
8

The derivative of the density is given by^13^
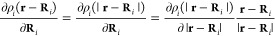
9

In CP2K the atomic densities ρ_*i*_ are calculated by performing a DFT calculation on the isolated atoms, and fitting a minimal Gaussian basis set to this density. As such, the radial derivatives are known analytically. The calculation of the atomic densities and their derivatives is performed only once per atomic species, and therefore the computational cost is negligible.

To both ensure numerical stability and to further reduce the computational cost, an adjustable cutoff is introduced for the denominator of eq 6. When the total promolecular density is smaller than 1e–12e the weight function is set to zero. Similar numerical cutoffs can be found in other implementations of CDFT based on Hirshfeld partitioning of the electron density.^22^ We have verified that the total energy and forces are insensitive to this choice of cutoff.

A simple test of the implementation of the constraint force (eq 5) and Hirshfeld partitioning (eqs 6–9) can be performed by checking that the total force (eq 4) is equal to the force calculated from finite differences of the minimized energy functional *W*[ρ, *V*] (eq 3) subject to the density constraint eq 2. Such a comparison is performed for the helium dimer He_2_^+^, shown in Supporting Information Figure 1. The difference in the force obtained from eq 4 and the finite difference calculation is 4.70 × 10^–5^ H/Bohr, similar to that obtained from other CDFT implementations.^22^

## 3 Results

We present validation and benchmarking of the CDFT force implementation for both geometry optimization (Section 3.1) and molecular dynamics (Section 3.2), for a variety of systems. Through considering a wider selection of systems than previous work, we are also able to discuss the general reliability of condensed phase CDFT calculations (Section 3.4).

### 3.1 CDFT Geometry Optimization

#### 3.1.1 (H_2_O)_2_^+^ in a Vacuum

Charged dimers or molecular clusters are a well-known problem for standard DFT functionals.^3^ The electron delocalization error tends to favor charge delocalization over charge localization, in particular for situations where both these states are energy degenerate in exact theory, e.g., in a charged molecular dimer at the dissociation limit. CDFT can be used to correct this error. In the following we consider the water dimer cation, creating the charge localized state H_2_O^+^–H_2_O by imposing a charge difference constraint of *N*_c_ = 1e between the donor (H_2_O) and acceptor (H_2_O^+^) regions using the Hirshfeld weight function eq 6. The constraint is converged until the residual error is less than 1 × 10^–4^e, with a wave function gradient of 1 × 10^–6^ H. Calculations are performed in a vacuum for a center of mass distance of 10 Å, with the PBE-D3 functional.^28,29^ Geometry optimization is converged until the residual forces are smaller than 0.02 eV/Å. Unless specified otherwise these values were used for all systems studied in this work.

Table 2 shows the DFT optimized geometries of the isolated H_2_O^+^ and H_2_O molecules, confirming that CDFT geometry optimization of the charge localized state H_2_O^+^–H_2_O reproduces these geometries for the large water–water separation of 10 Å, as it should do. Not surprisingly, DFT predicts that the excess hole is equally delocalized over both molecules and the geometry of the two molecules is the same, between the one for neutral water and the water radical cation, i.e. H_2_O^0.5+^–H_2_O^0.5+^. Similar results are found for CDFT-MD performed at 300 K (see the Supporting Information).

**Table 2:** Geometry Optimization of a Water Dimer (H_2_O)_2_^+^ in a Vacuum at a Distance of 10 Å^a^

	DFT (isolated)	DFT	CDFT
(O_1_–H_1_)^+^/Å	1.017	0.988	1.017
(O_1_–H_2_)^+^/Å	1.017	0.989	1.017
θ_HOH_^+^	108.51	105.91	108.50
(O_2_–H_3_)/Å	0.970	0.988	0.971
(O_2_–H_4_)/Å	0.970	0.985	0.971
θ_HOH_	104.17	105.95	103.94

a With the use of CDFT to form the charge localized state H_2_O^+^–H_2_O, the bond lengths and angles of the isolated H_2_O^+^ and H_2_O molecules are reproduced. In comparison, standard DFT predicts that the excess hole is equally delocalized over both molecules and the geometry of the two molecules is the same.

#### 3.1.2 Electron Transfer in Solid MgO

While CDFT is an established method for calculating electron transfer (ET) parameters in molecular systems,^25,27,30−32^ applications to condensed phase/periodic systems remain rare to date. A notable example, however, is the electron tunnelling between between charged oxygen vacancies (termed F-center defects) in MgO, previously calculated with a plane-wave implementation of CDFT in CPMD.^27,31^ Oxygen vacancies have been shown to exist in MgO in three possible charge states: *F*^0^, *F*^+^, and *F*^2+^, corresponding to the localization of two, one, or zero electrons at the defect site.^31^ The electron tunnelling process between defect sites *i* and *ii* is therefore written as
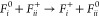
10

The ET process is modeled by removing two oxygen atoms at a separation *d* from a MgO rocksalt structure, while removing only one electron. With a total charge of +1 and a multiplicity of 2, a charge difference of *N*_c_ = 1e defined between the defect sites is used to form the diabatic states. The Hirshfeld weight function (eq 6) is defined as the 6 Mg atoms nearest to the respective defect site. The reorganization energy for this reaction is defined as
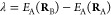
11where **R**_A_ and **R**_B_ are the optimized geometries in the diabatic states A and B. As the initial and final states are the same, the reorganization energy can be calculated as the vertical energy gap at the minimum of a diabatic state
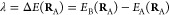
12

CDFT geometry optimizations of the diabatic states are performed using the PBE functional,^28^ with single point calculations of the reorganization energy also performed using the PBE0 functional^28,33,34^ with an optimally tuned truncated Coulomb potential.^35^ The latter functional was shown to reproduce the experimental MgO band gap of 7.2 eV.^27,36^ For Mg, the 2s, 2p, and 3s electrons and for O the 2s and 2p electrons are treated explicitly. Because of the very hard pseudopotential of Mg, a multigrid cutoff of 3000 Ry was used.

**Figure 1:**
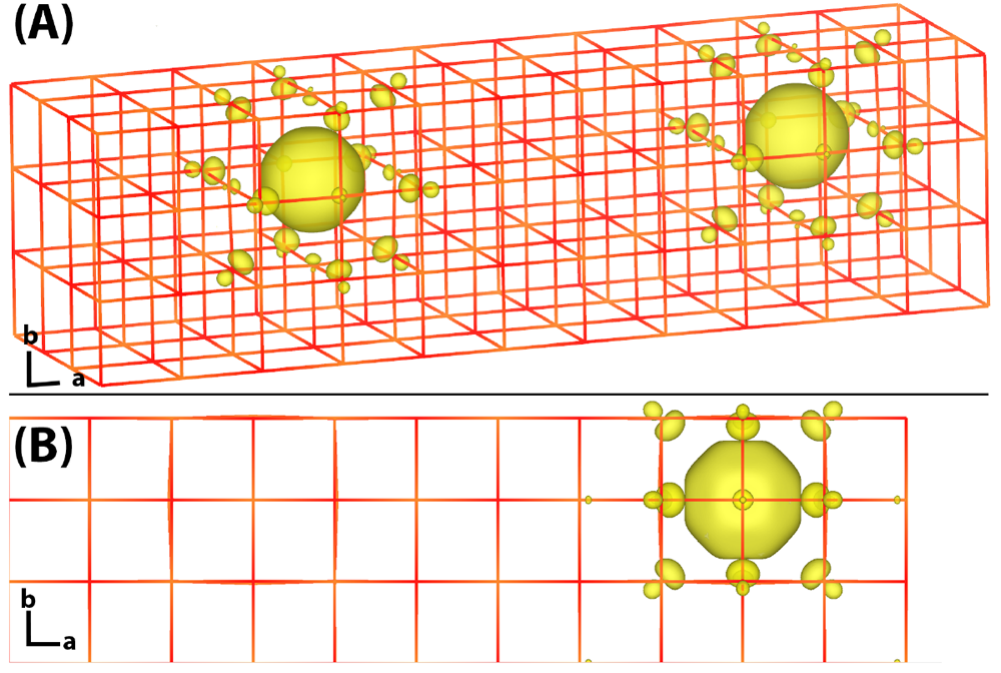
Oxygen defects in MgO. Excess spin density for (A) DFT adiabatic ground state and (B) CDFT diabatic state on the adiabatic ground state optimized geometry with a defect separation of 12.76 Å. The increase in spin density (yellow) is composed of a s-like function at the defect site and the p-orbitals of the surrounding oxygen atoms.

**Figure 2:**
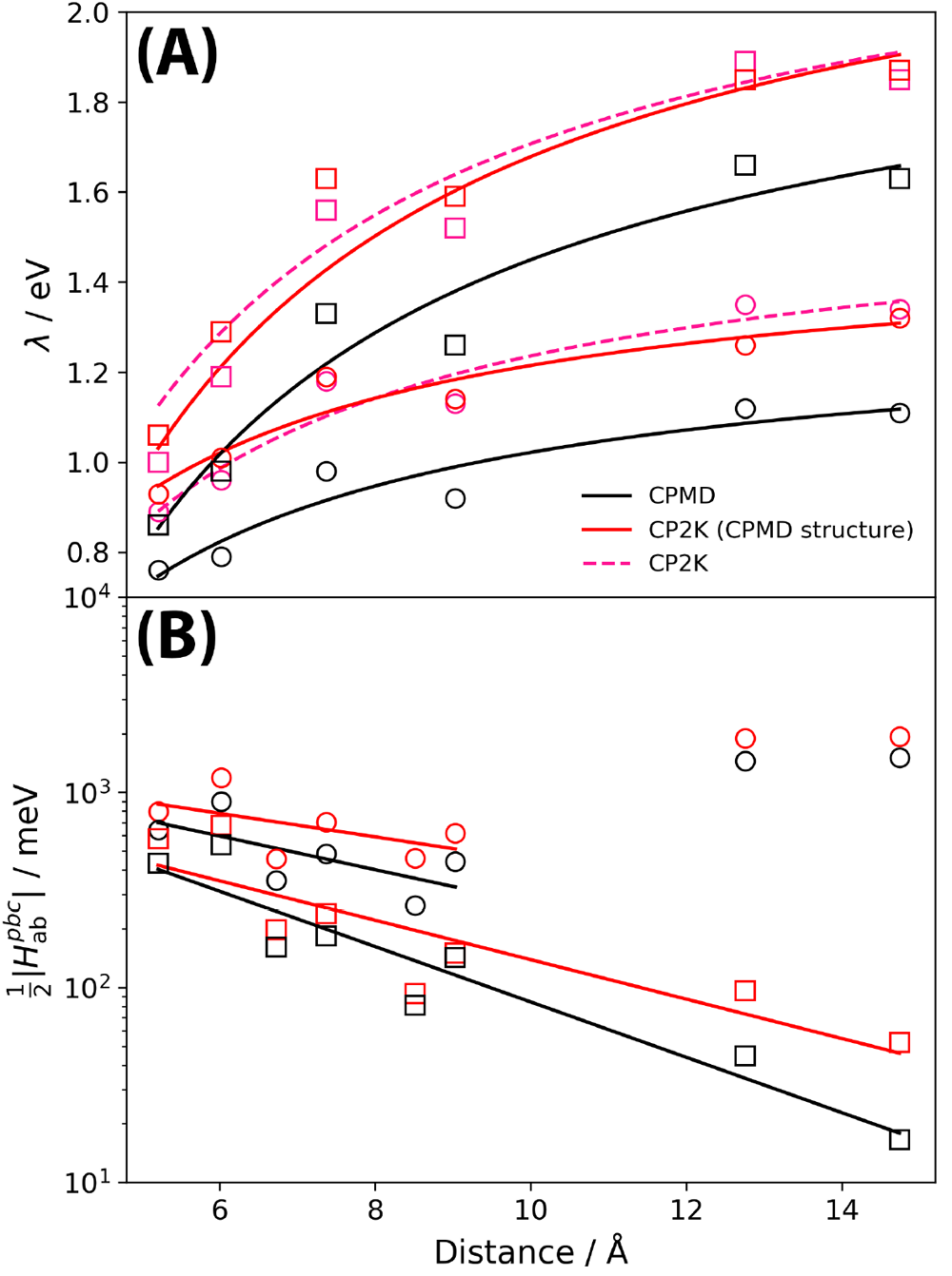
(A) Reorganization energies λ and (B) electronic couplings  obtained for tunnelling between oxygen defects in MgO. The black markers represent the CPMD reference values,^27^ red markers the CP2K values calculated using the CPMD structures, and pink markers the CP2K values from reoptimized structures. Results are shown for different percentages of Hartree–Fock exchange and for different defect separations. Circles represent PBE calculations, while squares represent PBE0 calculations. Best fits are indicated by solid and dashed lines.

Figure 1 shows an isosurface of excess spin density for the DFT adiabatic ground state, showing delocalization of the excess charge over both defect sites, and the CDFT diabatic state calculated for the same geometry with a charge difference of *N*_c_ = 1e defined between the defect sites. The results of geometry optimizing the diabatic state and calculating the vertical energy gap λ are shown in Figure 2. The corresponding reorganization energies (dashed lines) are very similar (MRUE = 4%) to the ones obtained from CP2K CDFT single point calculations on the CPMD CDFT optimized geometries (solid lines) giving reassurance to the present CDFT force implementation. The reorganization energies obtained from CP2K CDFT tend to be somewhat larger than they were reported for CPMD CDFT (MRUE = 22%), even if they are calculated on the same geometries. This difference is most likely related to the different functional form of the weight function *w* (eq 6) in the two implementations, Gaussian functions in CP2K and Slater functions in CPMD.^27,31^ Other differences, like the basis used for electronic structure calculations, could also contribute to the difference.

In addition to reorganization energies, we can also compare with the CPMD electronic couplings. The electronic coupling matrix elements between the initial and final ET states are calculated with CDFT^10^ on the transition state structures, approximated by the DFT adiabatic ground state where the electron hole is delocalized over both defects. The supercell size and defect separation were chosen such that in one direction the distance between the defects is equal to the distance to the periodic image of the defects, while the other directions are sufficiently large that periodic images in these directions have only a small effect.^27,31^ The finite size corrected electronic coupling *H*_ab_ is therefore equal to half of the coupling obtained in periodic boundary conditions *H*_ab_^pbc^ minus a correction term that accounts for the artificial contribution from the remaining periodic images
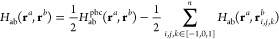
13where in this work the latter correction term is neglected to enable a direct comparison to the CPMD electronic couplings.

Figure 2 compares the electronic couplings calculated with CPMD and CP2K on the CPMD optimized geometries as a function of defect distance, with good agreement for defect distances of up to 10 Å (MRUE = 26%). At larger distances, both the PBE and PBE0 CP2K couplings are somewhat larger than reported for CPMD, resulting in a smaller exponential decay value for PBE0 of β = 0.47 ± 0.06 Å^–1^, compared to the one reported for CPMD couplings, 0.73 ± 0.10 Å^–1^.^27,31^ The overall MRUE error is reasonably small, 58%. For PBE, we find a smaller exponential decay value of β = 0.28 ± 0.10 consistent with CPMD β = 0.40 ± 0.22.

### 3.2 CDFT Molecular Dynamics

#### 3.2.1 H_2_^+^ in a Vacuum

An important consideration in any molecular dynamics calculation is total energy conservation. For CDFT-MD this can be particularly challenging as the constraint is introduced through an additional self-consistent field (SCF) loop, and as such both the DFT and CDFT SCF loops must be well converged in order to ensure total energy conservation.

**Figure 3:**
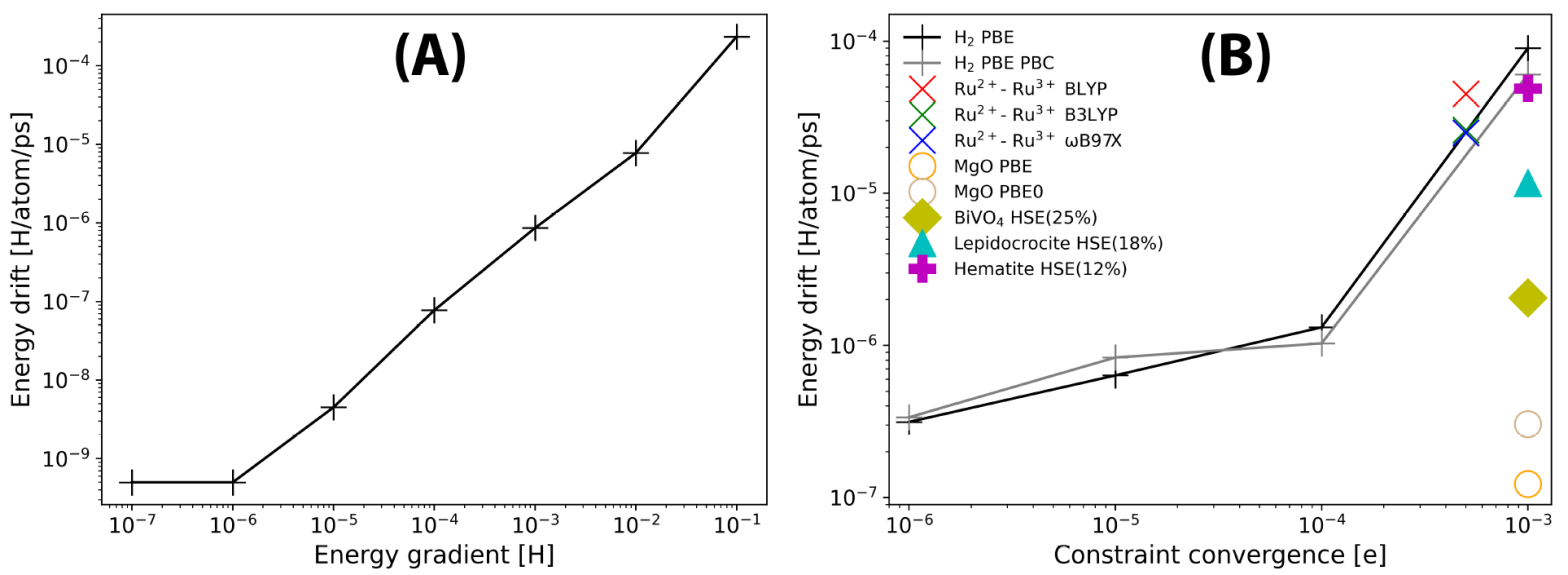
(A) Total energy conservation in DFT-MD of the hydrogen dimer H_2_^+^ as a function of the SCF convergence criterion, the largest gradient of the energy with respect to a change in molecular orbital coefficients. (B) Total energy conservation in CDFT-MD as a function of the constraint convergence. Solid markers for BiVO_4_, lepidocrocite and hematite denote systems where the energy drift for 1 ps has been extrapolated from 100 fs CDFT-MD. See Supporting Information Figure 8 for corresponding plots of energy drift against time.

The hydrogen dimer H_2_^+^ presents one of the simplest benchmarks for examining energy convergence, performed in a vacuum for a temperature of 300 K in the NVE ensemble with the PBE functional. Figure 3 shows the average drift of the conserved energy for both DFT-MD as a function of the SCF convergence criterion, and CDFT-MD as a function of the constraint convergence. The constraint is defined as a charge difference of *N*_c_ = 0.5e between the two hydrogen atoms. The resultant energy drift is less than 1e–6 H/atom/ps for a constraint convergence of 1 × 10^–6^e, the same as found in CPMD calculations for this system.^13^ For unconstrained DFT-MD of H_2_^+^, the energy drift is negligible for the chosen DFT convergence of 1 × 10^–5^, less than 1 × 10^–8^ H/atom/ps, and therefore the observed energy drift is introduced through the use of CDFT.

On average, the CDFT-MD calculations presented in this work are a factor of 3 times more expensive than corresponding DFT-MD calculations, consistent with other CDFT implementations.^13^ This additional cost is introduced by the CDFT SCF loop, with around 2–3 additional SCF cycles per MD step. See Supporting Information Figure 15 for the cost of CDFT-MD as a function of the constraint convergence.

#### 3.2.2 Excess Electrons and Holes in Oxide Materials

Also included in Figure 3 is the energy drift for CDFT-MD of MgO with a defect separation of 6 Å, constraining the charge difference over the defects sites as described in Section 3.1.2. Likely as a result of the well-defined oxygen defects with large reorganization energies, even for a loose constraint convergence of 1 × 10^–3^e total energy conservation below 1 × 10^–6^ H/atom/ps is achieved for both PBE and PBE0 CDFT-MD.

Energy drifts for CDFT-MD calculations for three further systems are shown in Figure 3: an excess electron in bismuth vanadate (BiVO_4_),^37^ an electron hole in lepidocrocite (γ-FeOOH),^38^ and an electron hole in hematite (α-Fe_2_O_3_).^38,39^ For each system, the starting structure is the geometry optimized charged ground state DFT structure of the excess electron or electron hole. A spin constraint is then used to constrain the spin moment of either the vanadium atom (bismuth vanadate) or the iron atom (lepidocrocite and hematite) where the polaron is localized to the spin moment of the geometry optimized structure. As such, the Lagrange multiplier (eq 3) is initially zero, and becomes finite during the CDFT-MD. Importantly, the use of CDFT-MD introduces a minimal additional energy drift in comparison with that for the use of DFT-MD. See Supporting Information Section 1.8 for additional information for these systems.

#### 3.2.3 Ru^2+^–Ru^3+^ in Aqueous Solution

For an example of condensed phase CDFT MD, we choose the previously studied Ru^2+^–Ru^3+^ electron self-exchange in aqueous solution.^13^ This is arguably one of the simplest electron self-exchange reactions in an aqueous solution. Both Ru ions are low-spin and coordinated by 6 water molecules in an octahedral geometry. The most significant difference between aqueous Ru^2+^ and Ru^3+^ is the Ru–O bond lengths, around 0.08 Å shorter in the oxidized state.^40^

**Figure 4:**
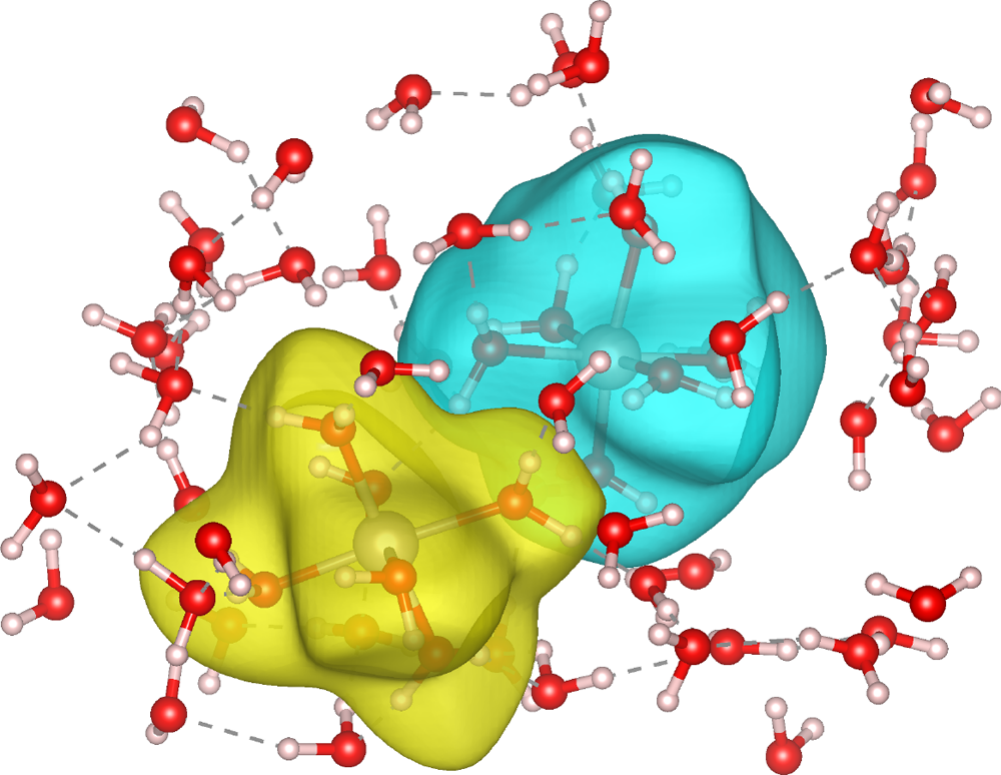
CDFT-MD of Ru^2+^–Ru^3+^ in aqueous solution. An isosurface of the weight function (eq 6) is shown, where the electron donating group Ru^2+^(H_2_O)_6_ is shown color coded yellow and the electron accepting group Ru^3+^(H_2_O)_6_ is shown color coded blue. The bonds between the 2 Ru ions and the 6 water molecules in their first solvation shell are shown explicitly.

Starting from the same initial structure from classical MD as the reference CPMD calculations,^13^ with 2 Ru ions and 63 water molecules, 1 ps of DFT-MD equilibration is performed with a time step of 0.96 fs in the NVT ensemble with a Nose–Hoover thermostat at 300 K, and a fixed Ru–Ru distance of 5.5 Å. Where possible, we use the same computational setup as the calculations in CPMD,^41,42^ including use of the BLYP functional.^43,44^ A charge difference constraint of *N*_c_ = 1e is defined between the electron donating and accepting groups, chosen as the Ru ion and the 6 water molecules in the first solvation shell: Ru^2+^(H_2_O)_6_ for the electron donating group and Ru^3+^(H_2_O)_6_ for the electron accepting group. The constraint is converged until the residual error is less than 5 × 10^–4^e. An isosurface of the weight function (eq 6) is shown in Figure 4.

The total linear drift of the conserved energy is shown in Figure 3, for both DFT-MD and CDFT-MD. The use of CDFT introduces minimal additional energy drift, with only a small increase from 4.0 × 10^–5^ H/atom/ps to 4.5 × 10^–5^ H/atom/ps. While this energy drift is reasonably large, it is smaller than that found in CPMD calculations of 9.7 × 10^–5^ H/atom/ps.^13^ See Supporting Information Figure 8 for a plot of the energy drift against time.

Following 1 ps of CDFT-MD equilibration, we find that the average absolute charge for the electron donating group Ru^2+^(H_2_O)_6_ is 0.47e and the electron accepting group Ru^3+^(H_2_O)_6_ is 1.47e. Only the charge difference between the two groups is constrained to 1, and as such the absolute charges are free to vary during the dynamics. These average charges are similar to those found from CPMD calculations, 0.52e and 1.52e.^13^ The remaining charge of 3.06e (2.96e from CPMD) is delocalized over the solvent.

While the average charges of the electron donating and accepting groups are similar between the CP2K and CPMD calculations, the geometries are different. For CP2K CDFT-MD the Ru–O bond lengths are on average 0.086 Å shorter in the oxidized state Ru^3+^(H_2_O)_6_ than Ru^2+^(H_2_O)_6_, in comparison to only 0.02 Å shorter for CPMD CDFT-MD.^13^ X-ray diffraction experiments performed on isolated ions in solution found that the average Ru–O bond lengths were 0.08 Å shorter in the oxidized state,^40^ consistent with unconstrained calculations performed in CPMD.^41,45^ However, without any experimental data available for an ion–ion distance of 5.5 Å it is not possible to determine which of the CPMD or CP2K CDFT-MD geometries are more accurate.

**Figure 5:**
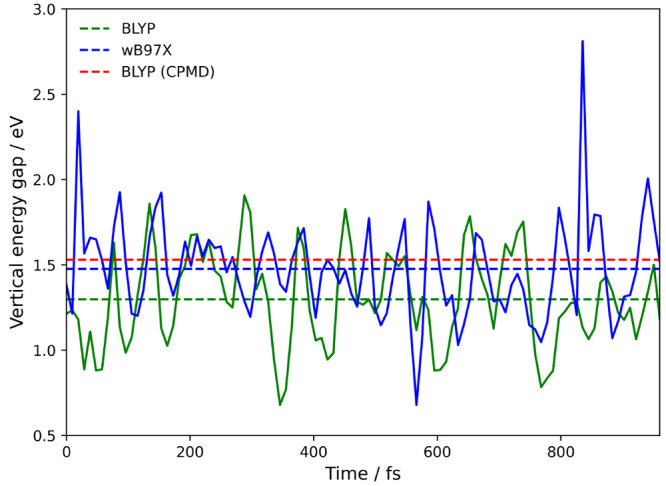
Vertical energy gap for the electron self-exchange reaction of Ru^2+^–Ru^3+^ in aqueous solution. Single point calculations are performed on 100 equally spaced structures sampled from 1 ps of CDFT-MD. The green dotted line shows the BLYP average of 1.30 ± 0.03 eV, the blue line the ωB97X average of 1.48 ± 0.08 and the red dotted line the CPMD value from Oberhofer et al. of 1.53 ± 0.06 eV.^13^

The CDFT simulation can be used to calculate the reorganization free energy for electron transfer between the two Ru ions. For self-exchange and assuming a linear response, it is simply equal to the thermal average of the vertical energy gap
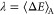
14where Δ*E* = *E*_B_ – *E*_A_, and the average is taken along a CDFT trajectory in diabatic state A. The vertical energy gap was sampled with 100 equally spaced single point calculations, shown in Figure 5, with an average ⟨Δ*E*⟩_A_ = 1.30 ± 0.03 eV, slightly smaller than the CPMD calculated value of 1.53 ± 0.06 eV.^13^ The error of the average due to the finite length of the trajectory is calculated from the difference of the vertical energy gap obtained from the first and second half of the trajectory.

With the increasing efficiency of computer codes and platforms, it is now possible to perform hybrid CDFT calculations on system sizes that would have been out of reach of the earlier CPMD work.^13^ In particular, we are able to perform CDFT-MD with B3LYP^46^ and the long-range corrected hybrid functional ωB97X.^47^ Following CDFT-MD equilibration, we find only a small increase in the average absolute charges of the Ru^3+^(H_2_O)_6_ and Ru^2+^(H_2_O)_6_ compared to charges obtained from BLYP CDFT-MD: +0.08e for B3LYP and +0.13e for ωB97X. The remaining charge of 2.92e and 2.82e remains delocalized over the solvent. Therefore, even these hybrid functionals are unable to prevent spurious charge delocalization across the solvent. Table 3 shows the average Ru–O bond lengths and vertical energy gap for BLYP, B3LYP and ωB97X. Similar to the charge, the reorganization energy increases only slightly: +0.12 eV for B3LYP and +0.18 eV for ωB97X.

**Table 3:** Average Ru–O Bond Lengths and Vertical Energy Gap (eq 14) for the Electron Self Exchange Reaction of Ru^2+^–Ru^3+^ in Aqueous Solution^a^

functional	average Ru–O (Å)	energy gap (eV)
BLYP	2.18, 2.10	1.30 ± 0.03
B3LYP	2.18, 2.08	1.42 ± 0.18
ωB97X	2.17, 2.07	1.48 ± 0.08
BLYP^13^	2.15, 2.13	1.53 ± 0.06

a The average of the 6 Ru^2+^–O and Ru^3+^–O bond lengths are calculated following 1 ps of CDFT-MD equilibration. The error of the vertical energy gap (eq 14) is calculated from the difference of the vertical energy gap obtained from the first and second half of the trajectory.

The reorganization energy calculated for the electron self-exchange reaction accounts for the 2 Ru-hexahydrates and the 51 water molecules solvating the electron transfer complex, neglecting the effects of higher solvation shells and the bulk solvent. Blumberger et al.^48^ calculated a finite size correction from classical MD with extrapolation to the limit of infinite dilution, resulting in a correction term of 0.09 eV.^13^ As such, the reorganization free energy of the infinitely diluted system for the BLYP, B3LYP, and ωB97X functionals is 1.30 + 0.09 = 1.39 eV, 1.42 + 0.09 = 1.51 eV, and 1.48 + 0.09 = 1.57 eV. Comparison to the experiment is challenging as a direct experimental measurement of the reorganization free energy is not available, and the experimental Ru–O bond lengths for the electron transfer complex at a distance of 5.5 Å are not known. A continuum study^49^ with a Ru–Ru distance of 6.5 Å reported a reorganization free energy of 1.95 eV, which fits well the experimental rate constant,^50^ and is expected to decrease to 1.75 eV for a Ru–Ru distance of 5.5 Å.^13^ In addition, under a number of assumptions, an experimental value of 2.0 eV has been reported.^50^

### 3.3 Charge Transfer in Organic Crystals

A useful application of CDFT in organic semiconductor research would be to calculate reorganization energies for charge transfer in organic semiconductors, including the full outer-sphere contribution from the periodic crystal which is usually presumed to be small and therefore neglected. However, it would be useful to check this assumption from case to case. Refined values for reorganization free energy would improve the accuracy of the parametrization of charge transport simulations, including, e.g., charge hopping and nonadiabatic molecular dynamics.^51−53^

**Figure 6:**
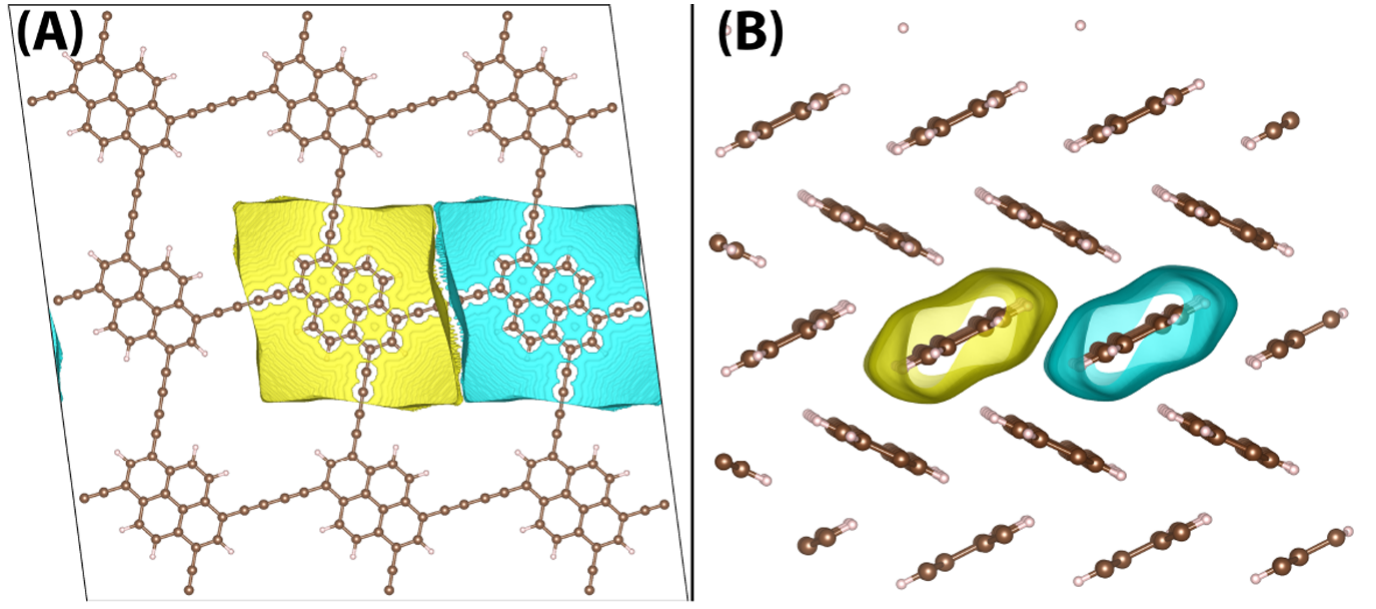
Weight function (eq 6) for hole transfer in two organic semiconductors: (A) 3 × 3 supercell of a pyrene 2D covalent organic framework (pyrene-COF) and (B) 3 × 2 × 1 supercell of pentacene.

Figure 6 shows the weight functions (eq 6) for hole transfer in two organic semiconductors: a 3 × 3 supercell of a pyrene 2D covalent organic framework (pyrene-COF),^54^ and a 3 × 2 × 1 supercell of pentacene. For both systems, the electron donating and accepting regions are defined as adjacent units or molecules. The reorganization energy for hole transfer in these systems should be calculated using eq 12, as the vertical energy gap at the minimum of a diabatic state. Geometry optimizing the diabatic state with PBE or HSE06 for either system results in unphysical distortions and even bond breaking during CDFT geometry optimization of the donor and acceptor groups. This shows that the localization of a full charge on a single COF unit or pentacene molecule within a crystalline environment does not correspond to a stable local minimum on the potential energy surface. Thus, we conclude that fully localized polarons do not exist in these materials and cannot be enforced using CDFT. In this respect, we note that previous nonadiabatic molecular dynamics simulations showed that polarons in crystalline pentacene are delocalized over 18 molecules on average at room temperature.^53^ At 0 K, corresponding to the present CDFT optimizations, the charge will occupy the fully delocalized state at the top of the valence band. The physical reason for the nonexistence of fully localized polaronic states is that reorganization energy is not sufficiently large in these materials compared to electronic couplings to support fully localized states, in stark contrast to, e.g., the F centers in the MgO system (Section 3.1.2) and the Ru^2+^–Ru^3+^ self-exchange reaction (Section 3.2.3).

### 3.4 Reliability of CDFT

CDFT is a powerful method for calculation of ET parameters, but as we have discussed in Section 3.3, no arbitrary charge constrained state can be constructed this way. A useful diagnostic tool to identify states that the DFT functional is not able to adequately describe is the IASD, eq 1. For a system with a single excess charge, the IASD should have a value of 1. Small deviations are to be expected. For example, the CDFT geometry optimizations of MgO in Section 3.1.2 have an average IASD of 1.05, and the CDFT-MD of Ru^2+^–Ru^3+^ in Section 3.2.3 have an average IASD of 1.09.

While neutral DFT calculations for the pyrene-COF and pentacene crystal in Section 3.3 have an IASD of 0.00 as expected, with the addition of an electron hole this increases to 1.18 for the pyrene-COF and 1.33 for the pentacene crystal. Using CDFT to localize the electron hole fully on a single unit or molecule raises the IASD to 1.46 (+0.28) and 1.55 (+0.22), with further increases during CDFT geometry optimization. See Supporting Information Figures 3 and 4 for the energy and IASD as a function of CDFT geometry optimization step. These large values of IASD indicate the breaking of electron pairs, as the DFT functional is not able to adequately describe the charged states. This is particularly problematic for CDFT, where the transfer of fractions of electrons from donor to acceptor can lead to electronic couplings that do not decay exponentially with distance.^55^ In the context of this work, we attribute symmetry breaking and the transfer of fractional electrons to the formation of an unphysical diabatic state that the DFT functional is not able to adequately describe.

## 4 Conclusion

In this work, we have provided an extension to CDFT in a popular DFT package CP2K, implementing the necessary force terms that arise from a constraint based on Hirshfeld partitioning of the electron density. The previously used Becke partitioning is prone to predict qualitatively incorrect atomic charges, as a result of dividing space equally among all atoms.

We have verified and benchmarked this new implementation against systems previously studied in a plane-wave implementation of CDFT, showing good agreement for both geometry optimization and molecular dynamics for electron tunnelling between oxygen defects in MgO^27^ and electron self-exchange in aqueous Ru^2+^–Ru^3+^.^13^ With the increasing efficiency of computer codes and platforms it is now possible to perform hybrid CDFT calculations on system sizes that would have been out of reach of the earlier CPMD work.^13^ In particular, we are able to perform CDFT-MD for electron transfer reactions in the condensed phase where both solute and solvent are treated at the hybrid or long-range corrected hybrid DFT level.

Consistent with previous work,^55^ we find that an IASD markedly larger than 1 is an indicator of systems for which CDFT calculations can be unreliable. With the exception of these pathological cases, we find that CDFT is a powerful tool for the calculation of electron transfer parameters at a reasonable computational cost. We expect the method to become valuable also for the simulation of electron transfer reactions across interfaces between different semiconductors or between semiconductors (e.g., oxides) and liquid solutions (e.g., water), thus becoming part of the toolbox for first-principles electrochemistry.^56^

## Supporting Information Available

Validation of forces from finite differences of the energy, additional information for MgO CDFT geometry optimization, additional information for pyrene-COF CDFT geometry optimization, additional information for pentacene CDFT geometry optimization, additional information for H_2_^+^ CDFT-MD, additional information for (H_2_O)_2_^+^ CDFT-MD, additional information for Ru^2+^–Ru^3+^ CDFT-MD, details of CDFT code (PDF)
