# Supplementary material for: Implementation and Validation of Constrained Density Functional Theory Forces in the CP2K Package
Source: J Chem Theory Comput. 2022 Jun 14;18(7):4438–46. doi: 10.1021/acs.jctc.2c00284 (PMC9281399; doi:10.1021/acs.jctc.2c00284)
Supplement: Supplementary file 1 — ct2c00284_si_001.pdf [file ct2c00284_si_001.pdf]

Supplementary Information: Implementation and validation of constrained density functional theory forces in the CP2K package

Christian S. Ahart,[†] Kevin M. Rosso,[‡] and Jochen Blumberger[*,†]

†*Department of Physics and Astronomy and Thomas Young Centre, University College London, London WC1E 6BT, UK*

‡*Pacific Northwest National Laboratory, Richland, Washington 99354, United States*

E-mail: j.blumberger@ucl.ac.uk

1 Supplementary Information

1.1 Validation of forces: He_2^+ in vacuum

Validation of the the CDFT force implementation can be performed by checking that these forces are equal to the forces calculated from finite differences of the energy. Such a comparison is performed for the helium dimer He_2^+, shown in Figure 1, demonstrating the self-consistency of the forces and energy. This verification is performed for both for real space interaction, and interaction via their periodic self-images. Average error without periodic boundary conditions is 4.7×10^{-5} Ha/Bohr, and under periodic boundary conditions 7.5×10^{-5} Ha/Bohr. These compare well to the PyCDFT reported value of 5×10^{-5} Ha difference.[1]

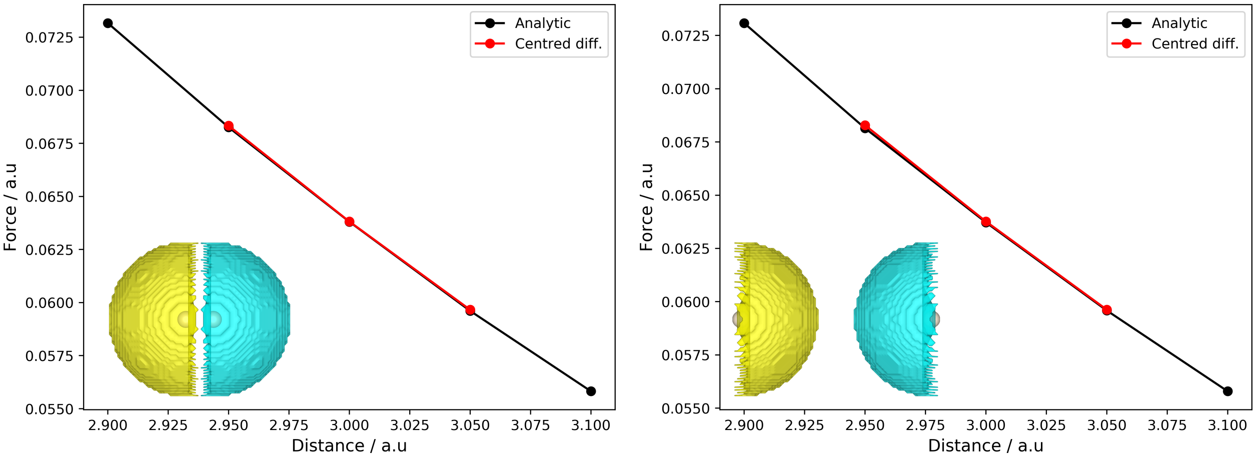

Figure 1: Verification of analytical forces against forces calculated from centred finite differences of the total energy for a helium dimer, both with (right) and without (left) periodic boundary conditions. For the system under periodic boundary conditions, the helium dimer interacts at the same distance except with their periodic image and therefore the resultant force is the same. An isosurface of the weight function is shown on the bottom left of each figure.

1.2 CDFT geometry optimisation: MgO

For completeness, we provide all the electronic couplings and reorganisation energies tabulated below. Values calculated using Becke charge partitioning are also included.

Table 1: Comparison of electronic coupling matrix elements obtained from CDFT calculations in CP2K with Becke weighting (BW), Hirshfeld weighting (HW) and reference values from CPMD. Results are shown for different percentages of Hartree-Fock exchange (HFX) and for different defect separations n.

% HFX	n	Cell	$\frac{1}{2}H_{\mathrm{ab}}^{\mathrm{pbc}}$ BW (meV)	$\frac{1}{2}H_{\mathrm{ab}}^{\mathrm{pbc}}$ HW (meV)	$\frac{1}{2}H_{\mathrm{ab}}^{\mathrm{pbc}\,2}$ (meV)
0	3	211-240-3	914.6	790.0	641.7
	4	110-192-4	1332.1	1189.1	899.2
	5	310-240-5	536.4	457.6	353.9
	6	111-144-6	882.4	704.3	485.5
	8	100-288-8	663.5	459.0	264.1
	9	100-288-9	874.4	610.3	440.8
	18	100-192-18	2245.1	1896.9	1447.5
	24	111-288-24	2246.5	1884.7	1507.9
25	3		613.4	569.4	432.1
	4		733.4	679.9	537.0
	5		173.6	201.6	161.3
	6		239.8	239.4	183.7
	8		92.6	93.8	81.0
	9		148.2	147.5	142.5
	18		145.3	89.2	44.6
	24		85.7	52.2	16.6

Table 2: Comparison of decay constant β obtained from CDFT calculations in CP2K with Becke weighting (BW), Hirshfeld weighting (HW) and reference values from CPMD.

% HFX	β BW ($\mathrm{\AA}^{-1}$)	β HW ($\mathrm{\AA}^{-1}$)	β CPMD^2 ($\mathrm{\AA}^{-1}$)
0	0.14	0.28	0.40
25	0.35	0.47	0.65

Table 3: Comparison of reorganisation energies obtained from CDFT calculations in CP2K with Becke weighting (BW), Hirshfeld weighting (HW) and reference values from CPMD. Results are shown for different percentages of Hartree-Fock exchange and for different defect separations n.

% HFX	n	Cell	λ BW (eV)	λ HW (eV)	λ_{opt} HW (eV)	λ^2 (eV)
0	3	211-240-3	0.94	0.89	0.93	0.76
	4	110-096-4	0.84	0.76	0.78	0.64
	4	110-240-4	1.07	0.96	1.01	0.79
	6	111-216-6	1.27	1.18	1.19	0.98
	9	110-144-9	1.25	1.13	1.14	0.92
	18	100-192-18	1.44	1.35	1.26	1.12
	24	111-288-24	1.37	1.34	1.32	1.11
25	3		1.07	1.00	1.06	0.86
	4		1.01	0.91	1.06	0.78
	4		1.34	1.19	1.29	0.98
	6		1.69	1.56	1.63	1.33
	9		1.69	1.52	1.59	1.26
	18		1.97	1.89	1.85	1.66
	24		1.87	1.85	1.87	1.63

The Integrated Absolute Spin Density is small for all MgO cells studied, and the energy gaps obtained between the CDFT states are only slightly larger than those from CPMD. The large energy gap for the 310-240-5 cell of 32 meV can be attributed to the challenging geometry, see Fig. 2.

Table 4: Integrated absolute spin density (IASD) and the difference in energy between CDFT states $|\Delta E|$ for all MgO cells studied in this work, with reference to available CPMD values.[2]

% HFX	n	Cell	IASD	$\lvert\Delta \mathrm{E}\rvert$(meV)	$\lvert\Delta \mathrm{E}\rvert$ CPMD[2] (meV)
0	3	211-240-3	1.04	2.0	
	4	110-192-4	1.05	0.9	
	5	310-240-5	1.04	32.2	
	6	111-144-6	1.04	12.6	
	8	100-288-8	1.04	3.2	
	9	100-288-9	1.04	4.7	
	18	100-192-18	1.04	10.6	
	24	111-288-24	1.04	6.45	
25	3		1.05	1.1	
	4		1.06	1.2	0.8
	5		1.05	38.7	28.6
	6		1.05	15.9	
	8		1.05	2.2	
	9		1.05	5.8	
	18		1.05	17.3	
	24		1.04	11.1	

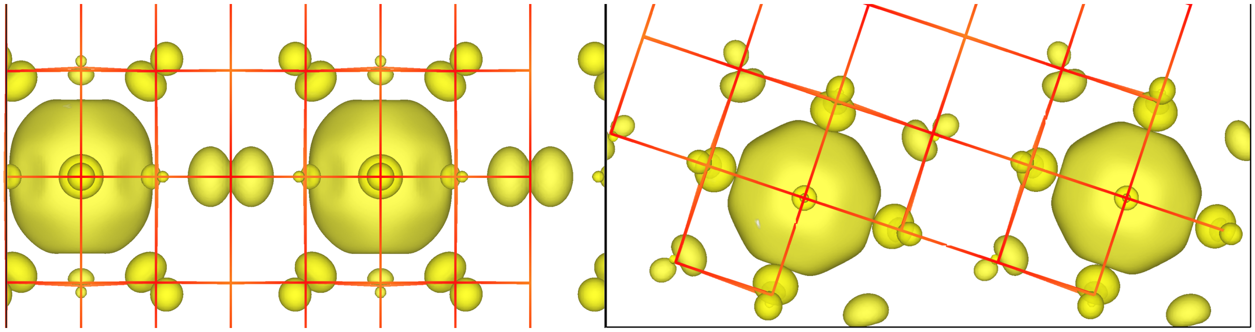

Figure 2: Excess spin density for ground state DFT optimised structures for the smallest CDFT energy difference cell 110-192-4 cell(left) and the largest energy difference cell 310-240-5 cell (right). It is clear that the larger energy difference is a result of the more challenging geometry optimisation.

1.3 CDFT geometry optimisation: 2D Pyrene COF

The usual process for calculating the reorganisation energy of organic molecules would be in the gas phase, however CDFT provides an ability to constrain the excess charge to a single unit in a periodic crystal and therefore account for the full outer-sphere reorganisation energy. We show an example of hole transfer in a 2D pyrene based covalent organic framework (COF), where the subsequent geometry optimisation diverges and leads to an increase in energy of 54 eV. Several different constraint definitions have been tested: the charge difference between two units shown below, the charge difference between one unit and the rest of the system, and an absolute charge constraint over one unit. These, in addition to constraints defined including or excluding the acetylene linkers, all lead to a large IASD and divergent geometry optimisation. This can be attributed to the effect of a too strong constraint, that the polaron in these materials is band-like[3] and attempting to constrain the excess charge to a single unit is an inappropriate choice of constraint as the underlying functional is not able to correctly describe the resulting electronic state.

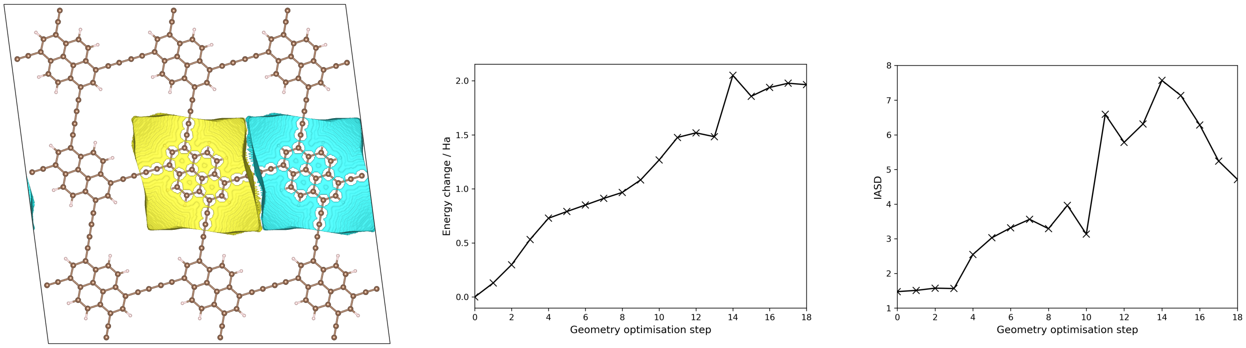

Figure 3: CDFT weight function defined as the charge difference between two units in a 3x3 2D pyrene cof (left), increase in energy per atom as a function of geometry optimisation step (middle) and increase in Integrated Absolute Spin Density as a function of geometry optimisation step (right). The large initial IASD of 1.46 and subsequent increase is an indication of fractional charge transfer, which generally occurs when the DFT functional is unable to appropriately describe the charge localised state.

1.4 CDFT geometry optimisation: Pentacene crystal

Similar to the pyrene-cof, it would be useful to calculate the reorganisation energy for a pentacene crystal accounting for the full outer-sphere reorganisation energy. Different constraints have been tested, constraining either the absolute charge or the charge difference between two pentacene molecules. In all cases, the IASD is large and the geometry optimisation diverges with an increase in energy of 30 eV after 13 geometry optimisation steps.

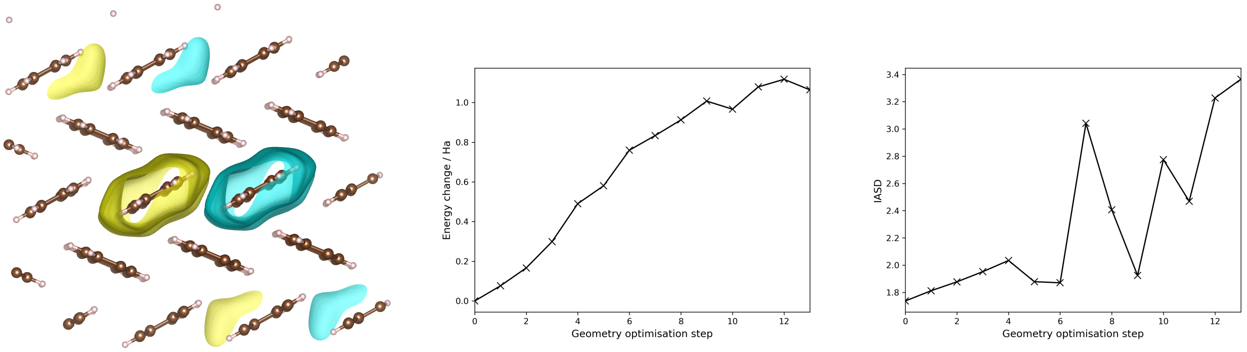

Figure 4: CDFT weight function defined as the charge difference between two pentacene molecules in a 3x2x1 pentacene cyrstal (left), increase in energy per atom as a function of geometry optimisation step (middle) and increase in Integrated Absolute Spin Density as a function of geometry optimisation step (right). The large initial IASD of 1.55 and subsequent increase is an indication of fractional charge transfer, which generally occurs when the DFT functional is unable to appropriately describe the charge localised state. Note that the repetition of the CDFT weight function (left) above and below the pentacene molecules is a visualisation artefact due to the non-cubic unit cell.

1.5 CDFT geometry optimisation: Pentacene in vacuum

Given the surprising results where the condensed phase CDFT geometry optimisation of pentacene fails, it is useful to confirm that CDFT works well for the pentacene dimer in vacuum. Here we confirm the exponential decay of the electronic coupling for both holes and electrons in a π-stacked pentacene dimer, despite their large IASD. This is consistent with the results published for the HAB11 dataset.[4–6]

Table 5: Electronic coupling for electrons and holes for the π-stacked pentacene dimer, confirming the exponential decay of the electronic coupling. Structures are taken from the HAB11 and HAB7- datasets.[4,5]

r / Å	H_{ab} hole / meV	H_{ab} electron / meV
3.5	638.9	708.3
4.0	228.8	250.9
4.5	98.0	111.2
5.0	40.2	49.3
β	3.66	3.52

Table 6: ISAD for hole and electron CDFT calculations of π-stacked pentacene dimer, with comparison to 5 Å CPMD CDFT calculation from the HAB11 dataset.[4]

r / Å	IASD hole	IASD electron
3.5	1.85	1.80
4.0	1.34	1.25
4.5	1.34	1.25
5.0	1.34, 1.38[4]	1.25

We further check the CDFT geometry optimisation of an excess hole for the π-stacked pentacene dimer in vacuum. For this example while the IASD increases, the total energy of the system decreases and the CDFT geometry optimisation succeeds with a reorganisation energy of 0.44 eV. This highlights a particular sensitivity of CDFT to condensed phase calculations, and suggests that the IASD is not always a reliable indicator for the breakdown of CDFT. It is possible however that CDFT only works for this system by fortuitous cancellation of errors, as described by the work of Van Voorhis and co-workers.[7]

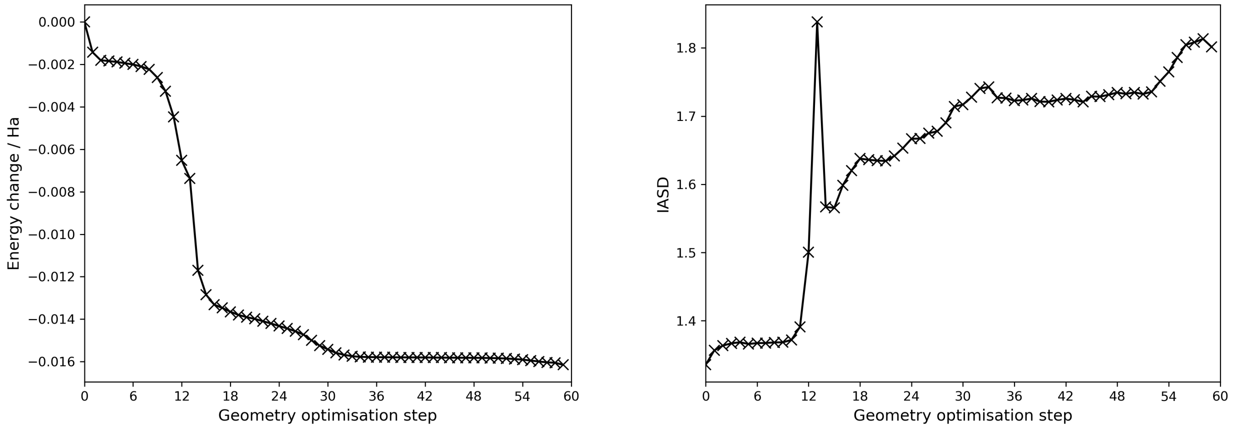

Figure 5: Decrease in total energy as a function of geometry optimisation step (left) and increase in Integrated Absolute Spin Density as a function of geometry optimisation step (right). Despite the large initial IASD of 1.34 and subsequent increase, the total energy decreases and the geometry optimisation converges.

1.6 CDFT-MD: H_2^+ and MgO

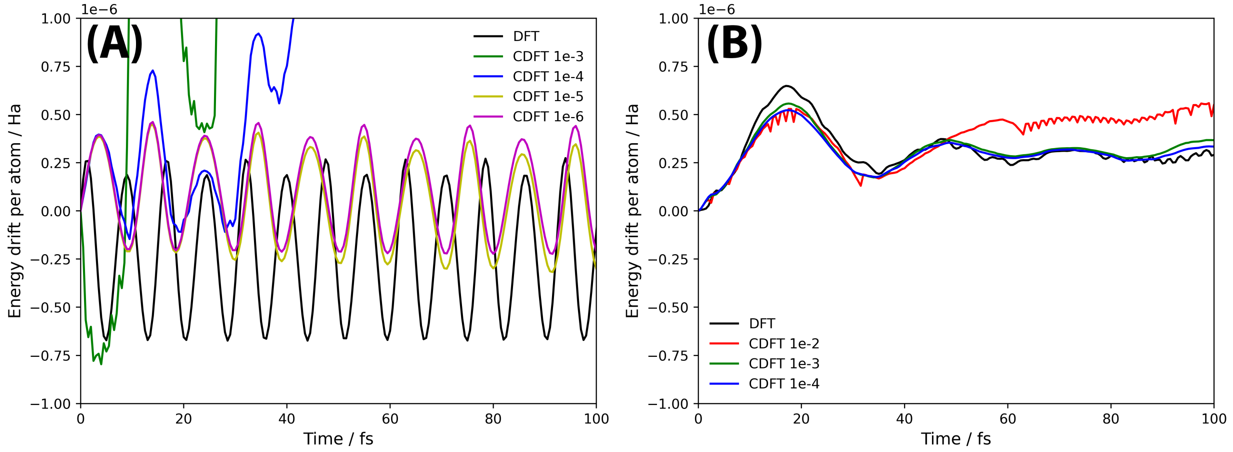

Figure 6: Energy drift as a function of time for CDFT-MD of (A) H_2^+ and (B) MgO with a defect separation of 6 Å.

1.7 CDFT-MD: H_2O^+-H_2O in vacuum

Alternative Table 2 from main text, with added column providing bond lengths and angles from CDFT-MD simulation of water dimer in vacuum. While not statistically converged, these values agree well with the geometry optimised results.

Table 7: CDFT geometry optimisation and molecular dynamics of charge localised state H_2O^+-H_2O. With the use of CDFT, the bond lengths and angles of the isolated H_2O^+ and H_2O molecules are formed.

	DFT (isolated)	DFT OPT	CDFT OPT	CDFT MD
$(O_1 - H_1)^+$ / Å	1.02	0.97	1.02	1.02
$(O_1 - H_2)^+$ / Å	1.02	0.96	1.02	1.02
θ^+_{HOH}	108.5	104.2	108.5	107.6
$(O_2 - H_3)$ / Å	0.97	0.97	0.97	0.98
$(O_2 - H_4)$ / Å	0.97	0.97	0.97	0.97
θ_{HOH}	104.2	104.4	103.9	102.0

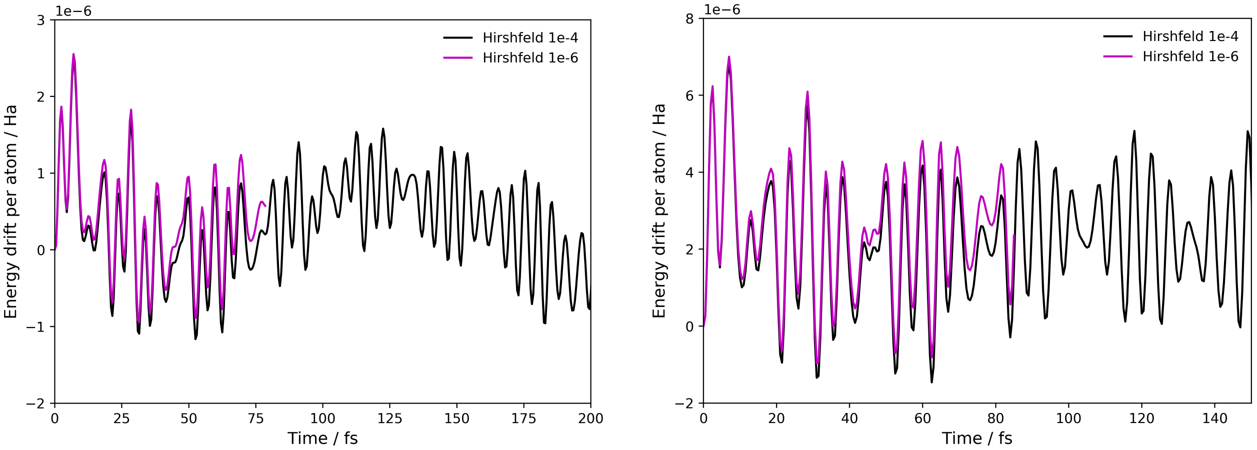

Figure 7: Energy drift as a function of time for H_2O^+-H_2O in vacuum at 100 K (left) and 300 K (right) during which bond lengths and angles were calculated. Black line is for a CDFT convergence of 1×10^{-4} and magenta line is for 1×10^{-6}e. Minimal energy drift is observed in both calculations.

1.8 CDFT-MD: Excess electrons and holes in oxide materials

As the use of condensed phase CDFT-MD is rare in the literature, we have also presented examples of CDFT-MD for: an excess electron in bismuth vanadate ($BiVO_4$),[8] an electron hole in lepidocrocite (γ−FeOOH)[9] and an electron hole in hematite (Fe_2O_3).[9,10] The HSE06 functional is used for all systems, with the percentage of Hartree-Fock exchange (HFX) optimised for each material: 25% for bismuth vanadate, 18% for lepidocrocite and 12% for hematite. The bismuth vanadate calculations are for a 1x2x1 supercell, the lepidocrocite calculations are for a 3x1x3 supercell and the hematite calculations are for a 2x2x1 supercell. Further details regarding the computational setup and polaron structures for lepidocrocite and hematite can be found in references 9 and 10.

For each system, the electron or electron hole polaron is localised on a single atom as indicated by a large change in spin moment from Hirshfeld analysis. As such, we use a spin constraint to constrain the spin moment of the atom where the polaron localises to the spin moment of the geometry optimised charged ground state: 0.91 for the vanadium atom in bismuth vanadate, -3.59 for the iron atom in lepidocrocite and -3.29 for the iron atom in hematite. Figure 8 compares the drift of the conserved energy as well as the value of the Lagrange multiplier as a function of time for these systems.

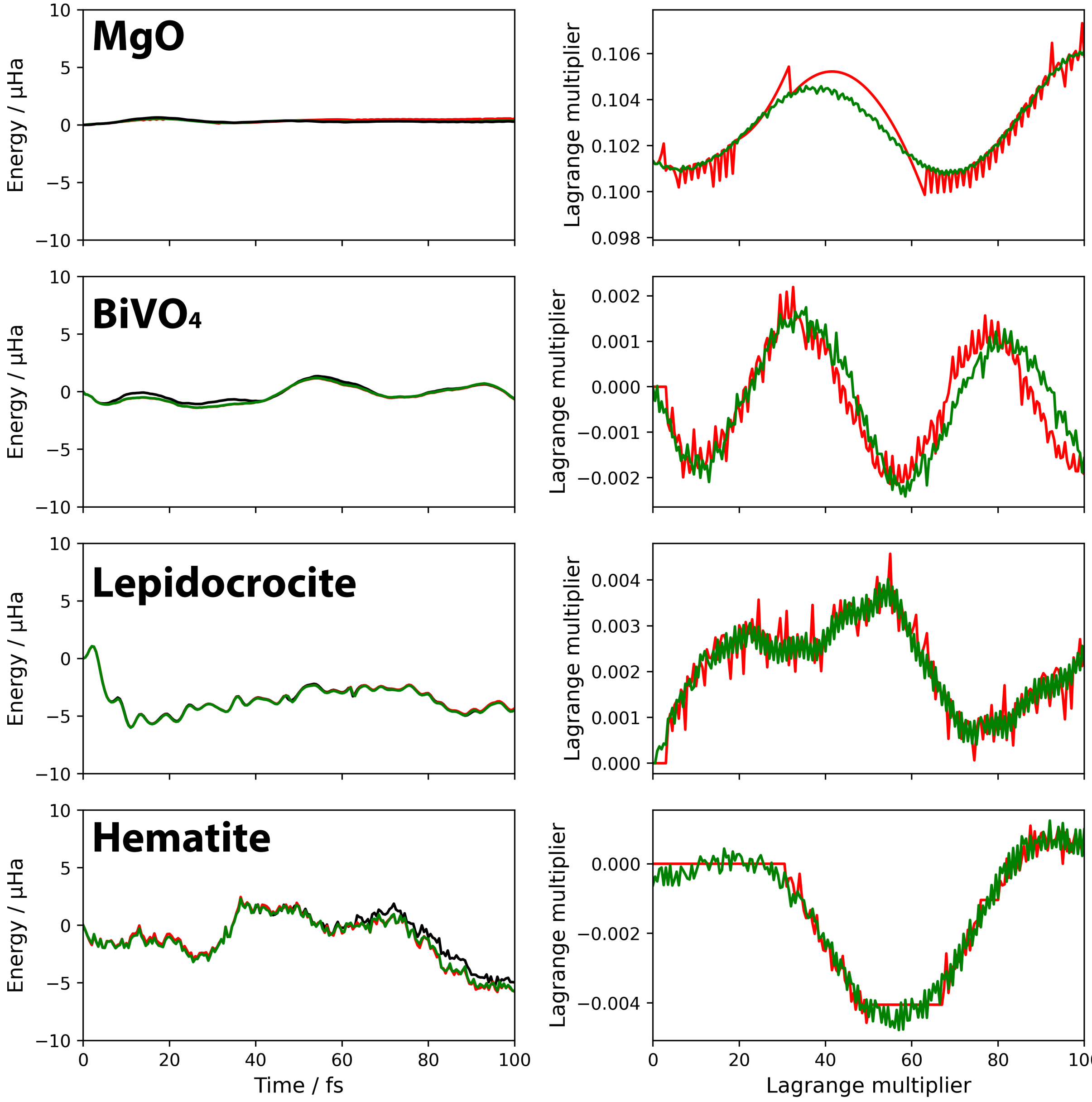

Figure 8: Drift of the conserved energy as well as the Lagrange multiplier for CDFT-MD calculations performed for: oxygen defects in MgO, an electron polaron in bismuth vanadate $BiVO_4$, a hole polaron in lepidocrocite and a hole polaron in hematite. Importantly, the use of CDFT-MD introduces minimal additional energy drift in comparison to DFT-MD. Black line represents DFT-MD, red line CDFT-MD with a constraint convergence of $1 \times 10^{-2}e$ and green line a constraint convergence of $1 \times 10^{-3}e$.

1.9 CDFT-MD: Ru^{2+}-Ru^{3+} in aqueous solution (BLYP)

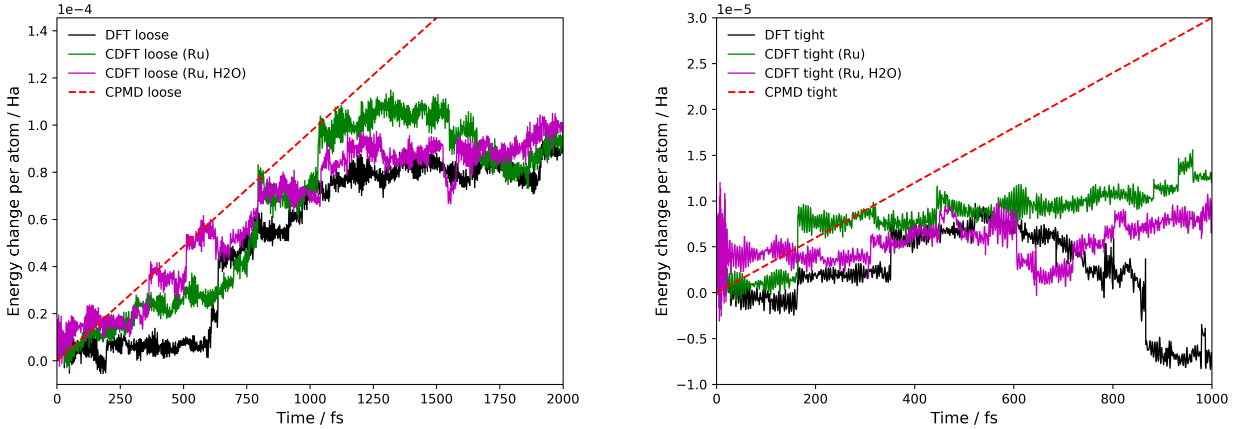

Figure 9: Energy drift as a function of time for the loose CDFT convergence criteria of $5 \times 10^{-4}e$ and a timestep of 0.98 fs (left), and a tighter convergence criteria of $5 \times 10^{-5}e$ and a timestep of 0.48 fs (right). The black line shows the energy drift of an equivalent DFT calculation, purple line a CDFT calculation where the weight function includes both the groups $Ru^{2+}(H_2O)_6$ and $Ru^{3+}(H_2O)_6$, the green line where the weight function includes only the Ru^{2+} and Ru^{3+}, and the red line a linear interpolation of the CPMD value. For both sets of calculations the CP2K energy drift is lower than the CPMD energy drift, and is only slighter higher than that of the underlying DFT calculation.

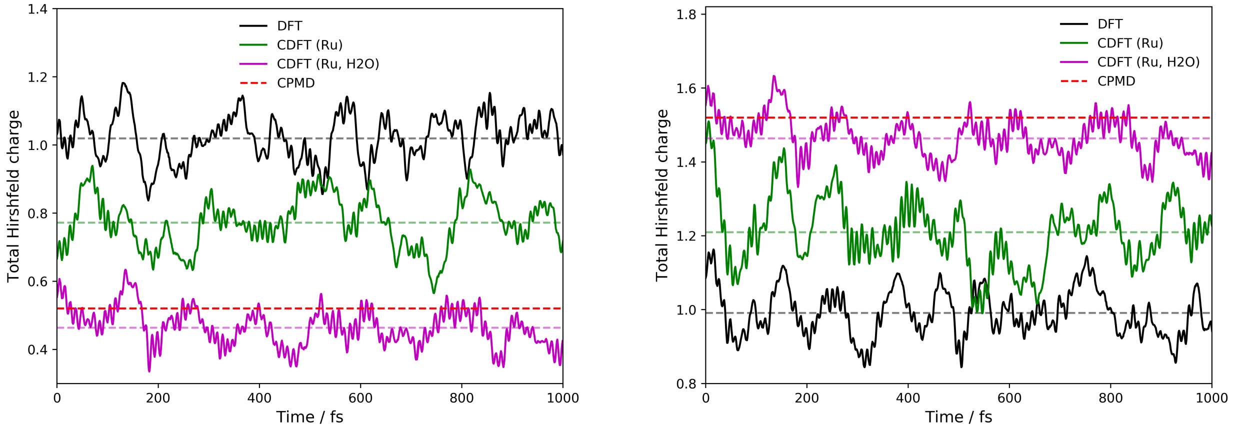

Figure 10: Average Hirshfeld charge for electron donating group $Ru^{2+}(H_2O)_6$ (left) and electron accepting group $Ru^{3+}(H_2O)_6$ (right).

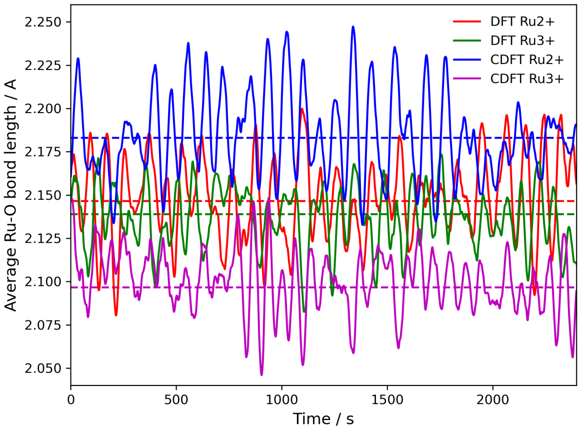

Figure 11: Average Ru-O bond lengths for Ru^{2+} and Ru^{3+} as a function of time for DFT-MD and CDFT-MD. Following 1ps of equilibration, the average Ru^{2+}-O bond lengths are: 2.15 Å (DFT), 2.18 Å (CDFT) and average Ru^{3+}-O bond lengths are: 2.14 Å(DFT), 2.10 Å(CDFT).

1.10 CDFT-MD: Ru^{2+}-Ru^{3+} in aqueous solution (BLYP, B3LYP, ωB97X)

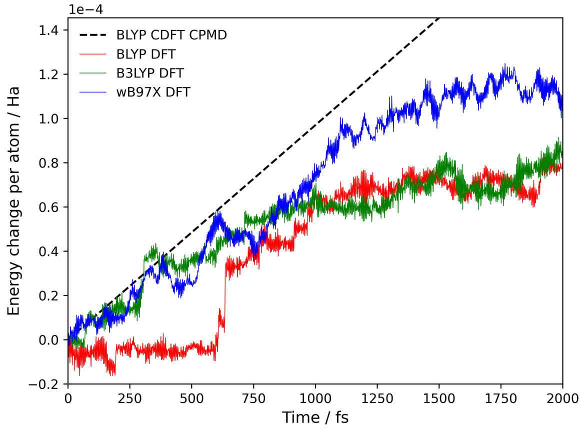

Figure 12: Energy drift as a function of time for the loose CDFT convergence criteria of 5×10^{-4}e and a timestep of 0.98 fs for DFT (left) and CDFT (right).

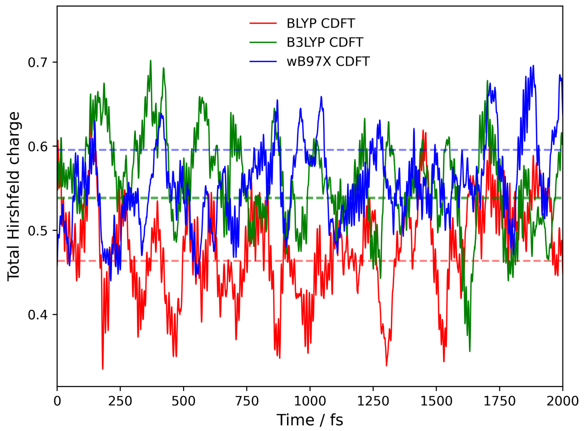

Figure 13: Average Hirshfeld charge for electron donating group $Ru^{2+}(H_2O)_6$ (left) and electron accepting group $Ru^{3+}(H_2O)_6$ (right).

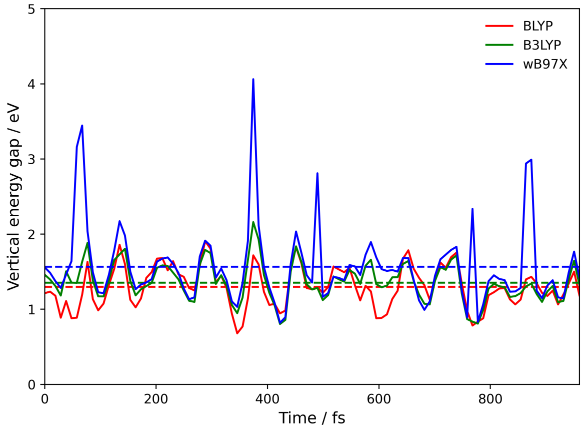

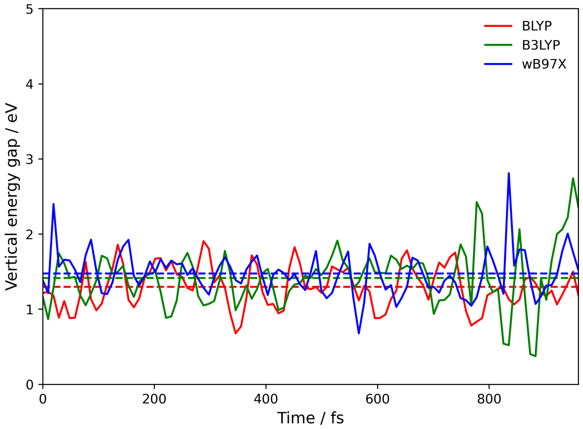

Figure 14: Vertical energy gap for the electron self exchange reaction of Ru^{2+}-Ru^{3+} in aqueous solution for single point calculations on BLYP structures (left) and on equilibrated structures (right).

1.11 Implementation of Hirshfeld CDFT

An implementation of CDFT forces using Hirshfeld partitioning of the electron density is now available in CP2K version 10.

Below, we present CDFT-MD energy conservation including data for a constraint convergence of $1 \times 10^{-2}e$ for condensed phase systems, and the time taken for an average CDFT-MD step relative to an equivalent DFT-MD step as a function of the constraint convergence. An important observation is that while hybrid DFT is more expensive than GGA DFT, the additional cost of CDFT is lower at the hybrid DFT level. This is likely a result of the under-binding of excess charge at the GGA level, which makes convergence of localised charges with CDFT more challenging and therefore the number of SCF steps is increased.

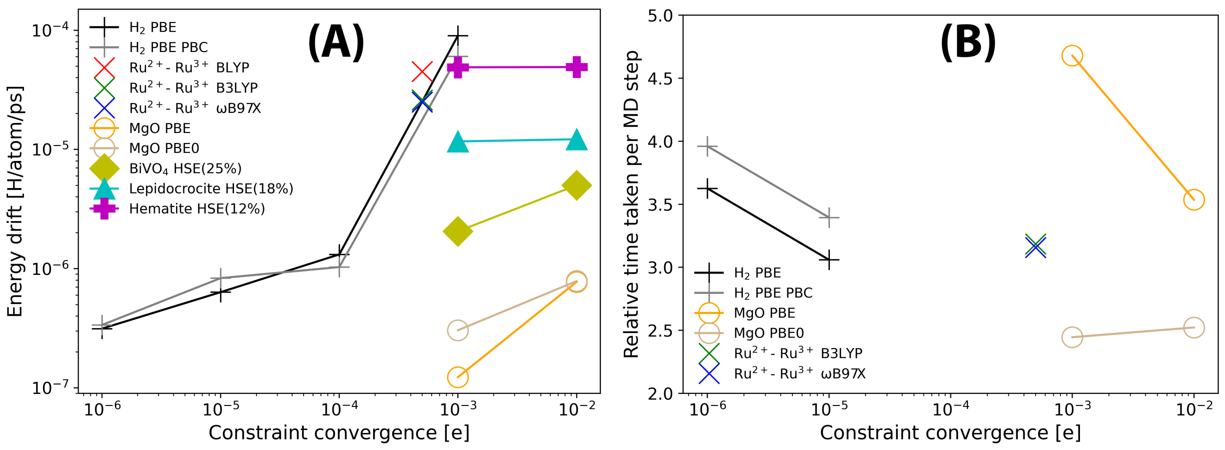

Figure 15: (A) Total energy conservation in CDFT-MD as a function of the constraint convergence. (B) Time taken for an average CDFT-MD step relative to an equivalent DFT-MD step. Solid markers for $BiVO_4$, lepidocrocite and hematite denote systems where the energy drift for 1 ps has been extrapolated from 100 fs CDFT-MD.

References

(1) Ma, H.; Wang, W.; Kim, S.; Cheng, M.-H. M.; Govoni, M.; Galli, G. PyCDFT: A Python package for constrained density functional theory. J. Comput. Chem. **2020**, 41, 1–9.

(2) Blumberger, J.; McKenna, K. P. Constrained density functional theory applied to electron tunnelling between defects in MgO. Phys. Chem. Chem. Phys. **2013**, 15, 2184–2196.

(3) Thomas, S.; Li, H.; Dasari, R. R.; Evans, A. M.; Castano, I.; Allen, T. G.; Reid, O. G.; Rumbles, G.; Dichtel, W. R.; Gianneschi, N. C.; Marder, S. R.; Coropceanu, V.; Brédas, J. L. Design and synthesis of two-dimensional covalent organic frameworks with four-arm cores: Prediction of remarkable ambipolar charge-transport properties. Mater. Horizons **2019**, 6, 1868–1876.

(4) Kubas, A.; Hoffmann, F.; Heck, A.; Oberhofer, H.; Elstner, M.; Blumberger, J. Electronic couplings for molecular charge transfer: Benchmarking CDFT, FODFT and FODFTB against high-level ab initio calculations. J. Chem. Phys. **2014**, 140, 104105.

(5) Kubas, A.; Gajdos, F.; Heck, A.; Oberhofer, H.; Elstner, M.; Blumberger, J. Electronic couplings for molecular charge transfer: Benchmarking CDFT, FODFT and FODFTB against high-level ab initio calculations. II. Phys. Chem. Chem. Phys. **2015**, 17, 14342–14354.

(6) Giannini, S.; Ziogos, O. G.; Carof, A.; Ellis, M.; Blumberger, J. Flickering Polarons Extending over Ten Nanometres Mediate Charge Transport in High-Mobility Organic Crystals ESI. Adv. Theory Simulations **2020**, 3, 1–35.

(7) Mavros, M. G.; Van Voorhis, T. Communication: CDFT-CI couplings can be unreliable when there is fractional charge transfer. J. Chem. Phys. **2015**, 143, 231102.

(8) Wiktor, J.; Ambrosio, F.; Pasquarello, A. Role of Polarons in Water Splitting: The Case of BiVO4. ACS Energy Lett. **2018**, 3, 1693–1697.

(9) Ahart, C. S.; Blumberger, J.; Rosso, K. M. Polaronic structure of excess electrons and holes for a series of bulk iron oxides. Phys. Chem. Chem. Phys. **2020**, 22, 10699–10709.

(10) Ahart, C. S.; Rosso, K. M.; Blumberger, J. Electron and Hole Mobilities in Bulk Hematite from Spin-Constrained Density Functional Theory. J. Am. Chem. Soc. **2022**,
